# A singular oral appliance to treat obstructive sleep apnea in CPAP non-adherent patients

**DOI:** 10.1590/2177-6709.25.5.044-050.oar

**Published:** 2020

**Authors:** Denise Fernandes Barbosa, Lilian Chrystiane Giannasi, Liege Maria Di Bisceglie Ferreira, Miguel Meira e Cruz, Marcelo Corrêa Alves, Fausto Berzin

**Affiliations:** 1 Private practice (Jundiaí/SP, Brazil).; 2 Universidade de Campinas, Faculdade de Odontologia de Piracicaba (Piracicaba/SP, Brazil).; 3 Universidade Estadual Paulista Júlio Mesquita Filho, Instituto de Ciência e Tecnologia (São José dos Campos/SP, Brazil).; 4 Sleep Unit, Centro Cardiovascular da Universidade de Lisboa, Lisbon School of Medicine, Lisbon, Portugal.; 5 Neuroimune Interface Research Lab, Faculdade São Leopoldo Mandic, Campinas-SP, Brazil.; 6 Universidade de São Paulo, Escola Superior de Agricultura Luiz de Queiroz (Piracicaba/SP, Brazil).

**Keywords:** Alternative treatment, Oral appliance therapy, Camper plane, Neuro-occlusal rehabilitation

## Abstract

**Introduction:**

The most prescribed treatment option for Obstructive Sleep Apnea (OSA) is CPAP; however, its adherence is limited. Oral Appliance therapy (OAT) is frequently an option or even an adjuvant, being the mandibular advancement Oral Appliance (OA_m_) the most used prescription. It modifies the upper airway, improving the airway patency. OA_m_ construction is based on the occlusal plane to disocclusion. In this study, the DIORS^®^ appliance was used, a singular OA_m_, based on Neuro-Occlusal Rehabilitation concepts, that uses Camper’s plane as a disocclusion reference, in order to achieve neuromuscular balance and functional stability.

**Objective:**

This study primarily aimed to assess the DIORS^®^ effectiveness in relation to clinical and polysomnographic outcomes. It was also evaluated if the use of DIORS^®^ is as effective as titrated CPAP to treat CPAP non-adherent patients.

**Methods:**

Twenty patients were included in this study. Objective and subjective clinical data were assessed at a sleep laboratory using all-night polysomnography, and Epworth Sleepiness Scale (ESS), taken at three moments: Baseline, CPAP titration, and using DIORS^®^. Analysis of respiratory parameters as apnea/hypopnea index (AHI), oxyhemoglobin saturation levels, the arousal index and daytime sleepiness were taken as criteria for a successful OAT.

**Results:**

Respiratory and arousal parameters improved in both therapies, while DIORS^®^ promoted a better ESS.

**Conclusion:**

Results from the present work support that DIORS^®^ is a viable and effective adjuvant therapy for patients with moderate to severe OSA non-adherent to CPAP.

## RESUMO

**Introdução:**

A opção mais indicada para tratamento da apneia obstrutiva do sono (AOS) é o CPAP; contudo, a aderência é limitada. A Terapia com Aparelho Oral (TAO) é, frequentemente, uma opção, ou mesmo um adjuvante. A prescrição mais utilizada é o Aparelho Oral de avanço mandibular (AO_m_). O AO_m_ modifica a via aérea superior, melhorando a patência do espaço aéreo. A construção do AO_m_ se baseia no plano de oclusão para desoclusão. No presente estudo, usamos o DIORS^®^, um AO_m_ diferente, baseado nos conceitos da Reabilitação Neuro-Oclusal (RNO), que utiliza o Plano de Camper como referência da desoclusão para alcançar o equilíbrio neuromuscular e estabilidade funcional.

**Objetivo:**

O presente estudo teve como objetivo principal abordar a eficácia do DIORS^®^, considerando-se os resultados clínicos e polissonográficos. Adicionalmente, foi avaliado, também, se o uso desse AO_m_ é tão eficaz quanto a titulação do CPAP para tratar pacientes com AOS não aderentes ao CPAP.

**Métodos:**

Vinte pacientes foram incluídos neste estudo. Dados clínicos objetivos e subjetivos foram avaliados em um laboratório de sono usando polissonografia de noite inteira e a Escala de Sonolência de Epworth (ESE) observando-se três momentos: inicial, titulação do CPAP e usando o DIORS^®^. Os critérios de sucesso da TAO foram assumidos pela análise dos parâmetros respiratórios como Índice de Apneia e Hipopneia (IAH) e níveis de saturação de oxi-hemoglobina, o índice de despertar e a sonolência diurna.

**Resultados:**

Em ambas as terapias, os parâmetros respiratórios e de despertares melhoraram. Adicionalmente, uma melhora na ESE foi alcançada com o DIORS^®^.

**Conclusão:**

Os resultados do presente trabalho apoiam que o DIORS^®^ é uma terapia adjuvante viável e bastante eficaz para pacientes com AOS moderada a grave não aderentes ao CPAP.

## INTRODUCTION

The most prescribed treatment option for Obstructive Sleep Apnea (OSA) is Continuous Positive Airway Pressure (CPAP), as this is considered the “gold standard” treatment^1^. However, adherence to CPAP is limited^2^ ^,^ ^3^ and therefore for non-adherent patients, Oral Appliance Therapy (OAT) is often an option or even an adjuvant treatment.^1^ ^,^ ^4^ ^-^ ^9^ The most common type of oral appliance is the mandibular advancement Oral Appliance (OA_m_). Several studies compare CPAP to OA_m_, and show that CPAP is more effective in reducing Apnea/Hypopnea Index (AHI).^3^ ^,^ ^5^ On the other hand, other studies found a lack of long-term relevant differences between CPAP and OA_m_ for mild to moderate OSA, when both treatment modalities are objectively titrated^3^. In addition, excessive sleepiness levels give rise to a primary and clinically important outcome in a sleep apnea patient’s follow-up, apparently showing no difference between OA_m_ and CPAP treatments.^10^ ^,^ ^11^ Recent studies have indicated that, despite the advantage of CPAP on AHI reduction, a high compliance to OA_m_, compared to CPAP^11^, leads to similar therapeutic effectiveness.

**Figure 1:**
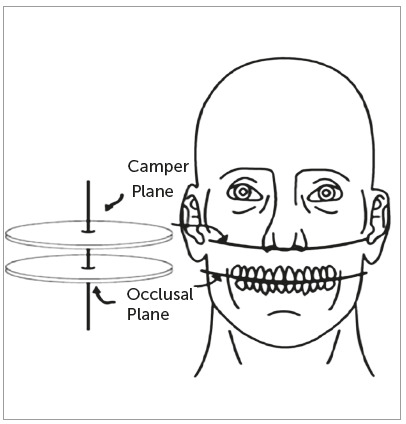
Frontal view of Camper’s plane (ala-tragus) and occlusal plane.

OA_m_ design from the new generation of oral appliances may impact on the therapeutic efficacy and effectiveness,^8^ ^,^ ^11^ ^-^ ^12^ with advanced main features, construction techniques, and the ability for individualization. Most OA_m_ use the Occlusal Plane (OP) orientation in the construction of dental disocclusion to mandible advancement. Historically, patient’s occlusal line has been assessed comparing the inclination to selected craniofacial reference lines. Some authors consider the Camper’s Plane (CP) the most suitable plane to orient the OP (Fig 1), based on fixed individual skull structures. Although neither enough long-term studies or authentic data are available advising on a single reliable landmark for the perfect OP, most have suggested CP for artificial orientation of OP.^13^ ^,^ ^14^

**Figure 2:**
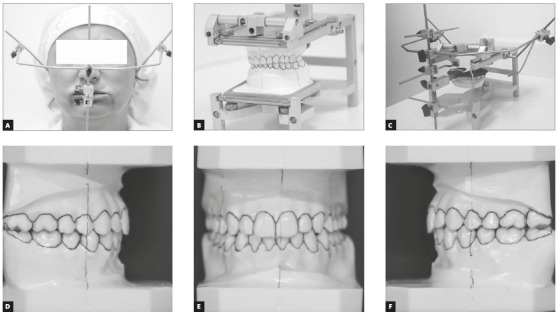
*Modus operandi* to make the gnathostatic model: A) gnathostatic facial arch; B) gnathostatic model construction; C) facial arch transferred to the Planas gnathostat to make the gnathostatic model; D) gnathostatic model, right side; E) gnathostatic model, frontal view; F) gnathostatic model, left side.

The OP can show differences in the orthogonal planes (sagittal, coronal and transversal), such as a unilateral masticatory function, generating skeletal asymmetries between the reference points of the orthogonal planes. Therefore, in the concepts of Neuro-Occlusal Rehabilitation (NOR),^15^ the main reference for a diagnosis is clinical examination of OP associated with CP to decide which treatment^16^ would bring neuromuscular balance and functional stability. Such diagnosis main tool is Gnathostatic Model (GM), observing the sagittal, coronal and transversal plane, to verify whether or not there is a CP and OP^15^ parallelism (Fig 2).

Anatomically, the tongue maintains several relationships with airway space;^17^ ^,^ ^18^ and so, with the hyoid bone and pharyngeal muscles.^19^ By changing mandible posture and tongue protrusion of an OSA patient, supra-hyoid muscles activity would also change, since it would clearly underline the role of tongue activity in maintaining upper airway patency in upper airway space.^20^

Respecting anatomic and physiologic conditions, and muscle origin and insertion to obtain functional balance, the DIORS^®^ (*Dispositivo Intra Oral Restaurador do Sono* ^*®*^ , Intra Oral Sleep Restoration Device) (Fig 2) was created.

Thus, the present study aimed at evaluating if the use of DIORS^®^, a model of OA_m_ using the CP for orientation to disocclusion, is sufficient to treat OSA patients not adhering to CPAP therapy.

## METHODS

### Study strategy

To compare the use of DIORS^®^ and CPAP therapy, CPAP non-adherent subjects were selected and the effects of DIORS^®^ were assessed. For this purpose, pre and post OAT subjective data, Epworth Sleepiness Scale (ESS), and polysomnography (PSG) objective data were compared. Three phases of the same patient were assessed: baseline, with CPAP titration (without adherence), and after DIORS^®^ adjustment (with adherence). The criteria for success in therapies were assessed with Arousal Index and respiratory parameters (Apnea/Hypopnea Index [AHI], Oxyhemoglobin Saturation [O_2_Sa], and daytime sleepiness). At the DIORS^®^ phase, the protocol was of 2-3 month for a change.

### Subjects

The Medical Ethics Committee of the *Faculdade de Medicina de Jundiaí* (SP, Brasil) (CAAE: 55049616.4.0000.5412 P.N. /1.529.053) approved this study that included patients attending the private clinic of one of the authors.

Anthropometric data of 20 subjects, 11 men and 9 women (Table 1), were consecutively collected. All patients non-adherent to CPAP treatment were indicated to adapt to OAT. 

**Table 1:** Mean / SD of baseline anthropometric data of all 20 patients (9 F and 11 M).

Variables	Mean (SD)
Age (years)	51,91 (12.66)
BMI (kg/height^2^)	28.58 (4.76)
Neck circumference (cm)	39.10 (4.39)
Waist circumference (cm)	102.05 (14.15)

SD = Standard Deviation; BMI = Body Mass Index; F = Male; M = Male.

Subjects selected should be adult, man or women, complaining of snoring, sleepiness, choking, with at least 8 teeth per dental arch, with positive diagnosis for OSA by PSG. Data was collected from 7 mild (5-15 ev./h), 8 moderate (16-30 ev./h) and 5 severe (>30 ev./h) OSA subjects. This study excluded patients without all PSG (baseline, CPAP titration and DIORS^®^ advancement), having mandibular advancement of less than 5 mm, a mandibular opening of less than 35 mm, tooth decay, extensive periodontal disease, predominant central sleep apnea, or muscle/joint pain.

### Questionnaires

At a sleep laboratory, the Epworth Sleepiness Scale (ESS) was used to evaluate subjective daytime sleepiness, taken at three moments: baseline, CPAP and DIORS^®^ titration. During the follow-up, partner and patient were interviewed to measure snoring and adherence. That moment was also used to assess how safe and resistant is the DIORS^®^ material. The interview included the following questions: “*Are you using the DIORS* ^*®*^ *”*; “*Do you use the DIORS* ^*®*^ *all night?”*; “*Do you use the DIORS* ^*®*^ *every night of the week?”*; “*Is your partner snoring with the DIORS* ^*®*^ *?”*; “*Are you fully satisfied with the DIORS* ^*®*^ ?”; “*Has the DIORS* ^*®*^ *ever broken?”.*

### Polysomnography

At the sleep laboratory, each subject was assessed regarding all-night baseline PSG. PSGs with CPAP titration and DIORS^®^ were also assessed (2 to 3 months after OA_m_ therapy). For that, sleep specialist physicians used 28-channels Brain Wave II (PSG Neuro Virtual, Barueri/SP, Brazil) following the 2007 AASM Manual for Scoring Sleep^21^. The channels consisted of: Referential AC inputs (8 electroencephalographic [EEG], 2 electrooculogram [EOG], 3 auxiliary); Bipolar AC input (1 electromyogram [EMG], 1 electrocardiogram [ECG], 1 snore, 1 flow, 1 pressure, 1 oximetry, 2 efforts, 1 position, 1 LM and 2 Aux); and 3 DC input. Sleep stages (wake [W]: sleep stage 1 [N1], sleep stage 2 [N2], sleep stage 3 [N3], and sleep stage REM [R]). AHI was defined as the number of episodes of apnea plus episodes of hypopnea per hour of sleep. OSA was defined as AHI ≥ 5.

### Treatment outcome

No consensus has been reached on how criteria for success should be defined^22^. Then, three success criteria were established regarding elimination or decreasing of AHI symptoms: 1) Successful (AHI < 5/h); 2) Partly successful (at least 50% reduction in AHI, but AHI > 5/h; and 3) Failure (persisting clinical symptoms, and/or less than 50% reduction in baseline AHI). Symptoms, adhesion, and satisfaction with the use of DIORS^®^ were assessed by means of a questionnaire applied to patients and partners. 

### Protocol of oral appliance therapy

First, to build the GM, a detailed anamnesis was performed at the first appointment. Impressions of the dental arches and the face bow were took to construct the GM. To determine the constructive bite, a George Gauge bite fork™ (Space Maintainers Laboratories, Chatsworth, CA, USA) was used. A specialized dental technician built the custom-made OA_m_ with 65-75% maximum protrusion and a vertical opening of 3-4mm between incisor edges. The construction of the DIORS^®^ required two gypsum casts: one for the GM, and one for the working model. 

Then, at the second appointment, the DIORS^®^ was placed. From that point, incremental advances of 1 mm were weekly performed, and the reports of patients regarding their experience with the DIORS^®^ were also recorded. Such reports indicated a decrease in snoring, gasps, sleepiness, and/or based on physiological limitations. The efficacy of the DIORS^®^ was determined by using additional PSG with DIORS^®^ *in situ*, after at least 3 months.

### DIORS^®^ construction, disocclusion, and advancement mechanism^20^

The construction of this OA_m_ is based on the definition of an OA_m_ published by the AADSM^23^. Briefly, the DIORS^®^ presented in this study is significantly different because it creates disocclusion and a position that allows a mechanism of advancement positioned on the posterior 2/3 of the tongue, on the lingual surface of the teeth. 

**Figure 3:**
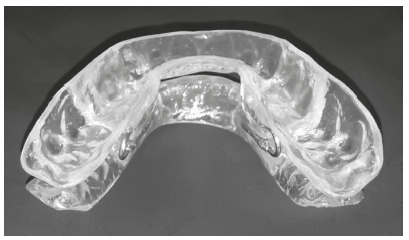
DIORS^®^ Brazilian OA_m_ (Intra Oral Sleep Restoring Device).

Disocclusion is guided by the CP through a device that replicates CP in the working model. Therefore, the DIORS^®^ performance promotes the protrusion of both the mandible and tongue (Fig 3). 

Additionally, this OA_m_ is duly patented with the National Institute of Intellectual Property (INPI, patent MU 202012025341-6), registered under numbers 904831639 (DIORS^®^) and 906.231.833 (DIORS^®^, *Dispositivo Intra Oral Restaurador do Sono* ^®^, or Intra Oral Sleep Restoring Device). 

### Statistical analysis

First, descriptive statistics was used to characterize the sample. Then, an ANOVA generalized linear mixed model was set to compare the three experimental conditions as repeated measures. Based on the Shapiro-Wilk test and asymmetry and kurtosis coefficients, the residual adherence to Gaussian distribution was assessed. The Tukey-Kramer test was applied as *post-hoc* test. 

Based on the results, state-of-the-art parametric techniques were used to describe a modern procedure to approach the issue. To assess the criteria for success and adherence patterns, chi-square was used, with a significance level of 5% in all tests.

## RESULTS

Table 2 shows the variables studied. Regarding the success criteria for therapies with AHI, CPAP and DIORS^®^ initial mean values were different (*p*= 0.0001) for the respiratory parameters (such as AHI, O_2_Sa _nadir_ and AI). Regarding daytime sleepiness, no difference was noticed between CPAP and DIORS^®^ at any sleep phase, except for TST-N2 mean (*p*= 0.001), which remained different at all phases, closer to normal in the DIORS^®^. Compared to the CPAP patients, DIORS^®^ patients reported reduced symptoms (*p*= 0.01) of subjective sleepiness. 

**Table 2:** Mean (SD). ANOVA (p-value) and Tukey’s test for variable mean in phases (baseline, after CPAP and titration), Average with equal superscript letters indicate no difference between them. Significance level was established as 5%.

Variables	Baseline	CPAP	DIORS^®^	p value
AHI/h	27.15 (27.90)^A^	3.55 (3.20) ^B^	6.16 (6.70) ^B^	0.0001
Sleep Latency N1 (min)	34.87 (27.94) ^A^	38.00 (21.82) ^A^	34.99 (24.08) ^A^	NS
TST (%) - N1	3.32 (3.21) ^A^	3.65 (3.30) ^B^	2.80 (0.71) ^AB^	NS
TST (%) - N2	59.05 (9.13) ^A^	51.56 (7.71) ^AB^	55.10 (5.54) ^B^	0.0015
TST (%) - N3	17.91 (6.96) ^A^	23.88 (7.86) ^A^	21.03 (5.67) ^A^	NS
REM (%)	19.71 (5.02) ^A^	20.91 (5.31) ^A^	21.10 (2.77) ^A^	NS
SE (%)	76.54 (12.84) ^A^	71.62 (12.20) ^A^	76.82 (10.42) ^A^	NS
O_2_Sa _mean_/h (%)	92.97 (1.78) ^B^	94.65 (1.60) ^A^	93.57 (1.77) ^B^	0.0005
O_2_Sa _nadir_/h (%)	82.68 (5.06) ^B^	88.45 (4.51) ^A^	87.45 (3.69) ^A^	0.0001
AI/h	23.93 (23.08) ^A^	6.56 (4.81) ^B^	6.55 (4.89) ^B^	0.0001
ESS	9.00 (5.77) ^AB^	9.06 (5.38) ^A^	7.22 (4.05) ^B^	0.0122
BMI (Kg/m^2^)	28.58 (4.76) ^AB^	28.69 (4.64) ^B^	29.34 (4.30) ^A^	0.044
CPAP titration (cm/H_2_O)	-	7.30 (1.92)	-	-
OA_m_ advancement (mm)	-	-	9.84 (2.67)	-

P<0.05. AHI= Apnea/Hypopnea Index; TST= Total Sleep Time; N1= sleep stage 1; N2=sleep stage 2; N3= sleep stage 3; REM= Rapid Eye Movement; SE = Sleep Efficiency; O_2_Sa= Oxyhemoglobin Saturation; AI= Arousal Index; ESS= Epworth Sleep Scale; OA_m_= Oral Appliance with mandibular advancement; CPAP= Continuous Positive Airway Pressure; NS- Non-Significant. A, B and AB= Superscript letters representing the Tukey test with a significantly different form.

Besides, Table 3 shows adherence in DIORS^®^ monitoring (*p*< 0,05). From the sample of individuals, three stopped using the DIORS^®^ due to bariatric surgery (n = 2) and dental treatment (n = 1).

**Table 3:** Affirmative descriptive data about follow-up of DIORS^®^ OA_m_ usage in 20 patients and partners interview (Chi-square test).

Questions	Yes - percentage (n)	p-value
Are you using the DIORS^®^?	85% (17)	0.002
Do you use the DIORS^®^ all night?	85% (17)	0.002
Do you use the DIORS^®^ every night of the week?	88.23% (15)	0.002
Is your partner snoring with the DIORS^®^?	58.8% (10)	0.467
Are you totally satisfied with the DIORS^®^?	88.23% (15)	0.002
Has the DIORS^®^ ever broken?	5% (1)	0.001

OA_m_= Oral Appliance with mandibular advancement.

## DISCUSSION

Although it is almost unknown^24^ to what extent the design of an OA_m_ impacts its efficacy, the present study shows that, compared to CPAP tritation, the DIORS^®^ may be a good alternative to CPAP non-adherent patients (Table 2), providing significant objective and subjective improvements.

Despite being considered a gold-standard therapy for moderate to severe OSA,^25^ ^,^ ^26^ efficiently reducing AHI,^24^ ^,^ ^26^ ^,^ ^27^ some authors^2^ criticize the CPAP concept due to its low adherence. Therefore, sleep physicians should monitor treatment adherence, and offer the oral appliance for OSA^27^ treatment to patients who do not adhere to CPAP therapy. This study demonstrates an alternative solution for that problem.

Many circumstances make AHI a controversial value, since it relies on the duration of events, temporal position of the events (NREM vs REM), or even especial conditions (chronic lung diseases, for instance). For that reason, additional parameters were used to better define severity in the present data sample. Hence, together with AHI and Oxygen Saturation, the results here discussed regarding the arousal index - this is considered an important parameter not only because it is an alternative criterion for scoring hypopneas, but also because it is important in the sum of total sleep duration, which is directly related to either sleepiness and cardiometabolic risk in OSA patients.

Although BMI has significantly increased in the DIORS^®^ therapy, actual parameters demonstrate a relevant improvement of respiratory (AHI and O_2_Sa) and AI (Table 2) parameters. As the main objective of the therapy, such results show good efficacy, as previously described for mild, moderate and severe OSA, supporting the use of DIORS^®^ based on clinical practice evidence.^1^ ^-^ ^3^ ^,^ ^5^ ^,^ ^8^ ^-^ ^11^ ^,^ ^17^ ^,^ ^20^ ^,^ ^25^ ^,^ ^27^ It also reestablished respiratory parameters to normal range (AHI < 5/h; *p*= 0.0001 and O_2_Sa_mean_> 93%; *p*= 0.0005), restored sleep (AI < 10/h; *p*= 0.0001), and reduced daytime sleepiness (ESS; *p*= 0.01).

The philosophy of the NOR^15^ ^,^ ^16^ and gnathological school^13^ ^,^ ^14^ advocate that the CP is the best reference plane for the occlusal rehabilitation because it promotes functional stability during stomatognathic functions. The DIORS^®^ design respects the same principles, in search for a better OAT adherence, with stability and neuromuscular balance, providing a good prognosis and successful treatment outcomes.

Previous studies^28^ ^,^ ^29^ report on objective and subjective adherence data, showing that the adherence of OA_m_ was about 83% when objectively evaluated, and 92% when subjectively assessed. In the present study, the DIORS^®^ had no objective measure to evaluate adherence, justifying the use of a questionnaire to obtain data of adherence and symptoms. In Table 3, due to patient comfort and tolerance,^27^ the monitoring data demonstrate 88.23% of DIORS^®^ adherence showing a slightly higher percentage than in previous OA_m_ studies.

Regarding DIORS^®^ safety, resistance, and durability^1.24^, this research showed its stability and efficacy. This OA_m_ was able to maintain airway patency at a therapeutic level of protrusion, being only one fracture noticed in the advancement mechanism, with 95% of safety and resistance.

Finally, for a better treatment outcome,^1^ ^,^ ^7^ ^,^ ^24^ ^,^ ^27^ ^,^ ^30^ a sleep doctor and a dental surgeon should compose the multidisciplinary team.

## CONCLUSION

The present study provides an opportunity to investigate the factors likely to determine how to manufacture the OA_m_ and how to assess if its design significantly changes neuromuscular responses, prognosis and treatment outcomes.
